# Cancer-Associated Fibroblasts-Orchestrated Immune Remodeling as a Key Driver in Liver Cancer

**DOI:** 10.32604/or.2026.083633

**Published:** 2026-10-28

**Authors:** Nunzia Porro, Silvia Marri, Francesca Salani, Fabio Marra, Laura Gragnani, Alessandra Gentilini

**Affiliations:** 1 Department of Experimental and Clinical Medicine, University of Florence, Florence, Italy; 2 Department of Translational Research and New Surgical and Medical Technologies, University of Pisa, Pisa, Italy

**Keywords:** Hepatocellular carcinoma, intrahepatic cholangiocarcinoma, cancer-associated fibroblasts, liver immunology, immune remodeling, immunotherapy

## Abstract

Hepatocellular carcinoma (HCC) and intrahepatic cholangiocarcinoma (iCCA) develop within a highly immunosuppressive tumor microenvironment (TME) characterized by chronic inflammation, extensive stromal remodeling, and impaired antitumor immunity. Among stromal components, cancer-associated fibroblasts (CAFs) have emerged as key regulators of tumor progression and immune homeostasis. This review provides an overview of CAF-mediated immune remodeling in primary liver cancers, with particular emphasis on CAF heterogeneity, CAF–immune cell interactions, and their impact on disease progression and therapeutic response. Increasing evidence indicates that CAFs orchestrate multiple aspects of the tumor immune microenvironment through the secretion of cytokines, chemokines, growth factors, and extracellular matrix components. These signals promote immune exclusion, suppress cytotoxic T-cell activity, and favor the accumulation of immunosuppressive cell populations, including regulatory T cells, myeloid-derived suppressor cells, and tumor-associated macrophages. Advances in single-cell and spatial transcriptomic technologies have revealed substantial CAF heterogeneity and identified distinct CAF subsets with specialized immunomodulatory functions. These findings have uncovered novel therapeutic opportunities, including CAF-targeted approaches, extracellular matrix normalization, and strategies aimed at reprogramming CAF plasticity. Overall, CAFs are central orchestrators of immune remodeling in liver cancer and contribute to the establishment of an immunosuppressive microenvironment that supports tumor progression and therapeutic resistance. Targeting CAF-driven stromal–immune interactions may enhance antitumor immunity and improve the efficacy of current and emerging immunotherapies in HCC and iCCA.

## 1 Introduction

Hepatocellular carcinoma (HCC) is a major global health challenge, ranking sixth in incidence and third in cancer-related mortality [1,2]. Unlike most solid tumors, its incidence and mortality have risen significantly, particularly in the United States. HCC accounts for ~80% of primary liver cancers, with most cases arising in patients with cirrhosis or fibrosis [3]. While Hepatitis B virus (HBV)- and Hepatitis C virus (HCV)-related HCC are expected to decline due to vaccination and antiviral therapies, metabolic dysfunction–associated fatty liver disease (MAFLD) is emerging as a leading cause driven by increasing obesity rates [4,5]. Despite well-defined risk factors, the genetic drivers of HCC remain poorly understood [6]. Early surveillance of high-risk populations is therefore crucial to improve outcomes and reduce socioeconomic burden [7,8].

Cholangiocarcinoma (CCA) arises from the malignant transformation of cholangiocytes or trans-differentiation of hepatocytes [4]. Its high clinical and molecular heterogeneity, together with an asymptomatic early phase, leads to delayed diagnosis and poor prognosis [4,9]. Risk factors include cirrhosis, HBV/HCV infection, MAFLD/Metabolic dysfunction–associated steatohepatitis (MASH), liver flukes, chronic biliary diseases (e.g., Primary sclerosing cholangitis [PSC]), and toxins, all associated with chronic inflammation [4,5]. Incidence and mortality are increasing globally, mainly due to intrahepatic CCA (iCCA) [10], and late diagnosis and limited treatment options result in poor 5-year survival (<12% for HCC and ~10% for CCA) [11].

Regarding early-stage HCC, curative options include resection, transplantation, and local therapies, whereas advanced disease is primarily managed with systemic treatments, including immune checkpoint inhibitor (ICI)-based combinations and tyrosine kinase inhibitors (TKIs) [12]. Atezolizumab plus bevacizumab and tremelimumab plus durvalumab are currently recommended first-line options for eligible patients, while lenvatinib or sorafenib may be considered when immunotherapy-based combinations are contraindicated [13,14].

CCA has a poor prognosis, with 5-year survival of 7–20% [15]. Early-stage disease is managed by surgery or, less commonly, transplantation, while advanced cases rely on chemotherapy, ICIs, locoregional, and targeted therapies. Cisplatin–gemcitabine plus durvalumab is the current standard systemic treatment, with targeted options for IDH1 and FGFR2 alterations and ICIs for MSI-high tumors [16]. However, treatment efficacy remains limited for many patients, highlighting the need for additional therapeutic strategies. The efficacy of immune checkpoint blockade (ICB) is strongly influenced by the tumor immune microenvironment (TIME), whose heterogeneity shapes tumor progression, immune evasion, and therapy response. Among stromal components, cancer-associated fibroblasts (CAFs) are key regulators of immune remodeling and major contributors to immunotherapy resistance in HCC and iCCA. While HCC is closely linked to chronic liver fibrosis [17], iCCA is not invariably associated with a pre-existing fibrotic background but exhibits a pronounced desmoplastic stroma rich in CAFs, similar to pancreatic and prostate cancers.

Currently, fibroblast-targeted strategies are largely unavailable. Although therapeutic advances have recently expanded treatment options for MASH with fibrosis, these approaches do not directly target activated fibroblasts or specifically promote fibrosis resolution [18,19]. Likewise, in CCA, currently available therapies do not specifically target CAFs or their mediators, partly reflecting the lack of established fibroblast-directed treatments [20]. CAFs promote tumor progression through secretion of soluble factors, extracellular matrix (ECM) remodeling, and interactions with other cells, driving proliferation, angiogenesis, immunosuppression, and chemoresistance [15]. Despite this, research has mainly focused on their fibrogenic role, overlooking their immunomodulatory functions. CAF enrichment is now recognized as a hallmark of progression, immune escape, and therapy resistance in both HCC and iCCA, highlighting them as promising yet underexplored therapeutic targets.

CAFs modulate the immune system by promoting tumor immune evasion and shaping responses to immunotherapy through immunosuppressive cytokines and checkpoint pathways, including TGF-β and PD-1/PD-L1 [21,22,23,24,25]. They also recruit myeloid-derived suppressor cells (MDSCs), induce protumoral phenotypes in neutrophils and macrophages, and contribute to CD8^+^ T-cell exclusion, affecting both innate and adaptive immunity [26,27,28,29,30,31]. This remodeling drives an “immune-excluded” TIME associated with resistance to checkpoint inhibitors in HCC and iCCA.

Although CAF targeting may provide diagnostic, prognostic, and therapeutic opportunities, conflicting evidence from preclinical models highlights their functional heterogeneity [32,33,34]. Recent single-cell and spatial transcriptomic studies have identified distinct CAF subsets with specific immunoregulatory roles, including antigen-presenting, myofibroblastic, and inflammatory CAFs [35,36,37,38,39,40].

Despite the growing number of studies investigating CAF biology in liver cancer, most available reviews have primarily focused on fibrosis, stromal remodeling, or individual tumor types. In contrast, recent advances in single-cell RNA sequencing (scRNA-seq) and spatial transcriptomics have revealed previously unrecognized CAF subsets with distinct immunoregulatory functions. Building on these advances, this review specifically examines CAF-driven immune remodeling across both HCC and CCA, integrating emerging evidence from single-cell and spatial transcriptomic studies within a unified TIME framework.

Given the unique TIME of liver cancers, understanding CAF-driven stromal and immune remodeling is essential to overcome immunotherapy resistance. Therefore, the aim of this review is to summarize the current knowledge on CAF biology and heterogeneity in HCC and CCA, with particular focus on their immunomodulatory functions, their interactions with immune and stromal components within the TIME, and emerging stromal- and immune-targeted therapeutic strategies that may improve patient outcomes and overcome resistance to current treatments. It is important to note that the understanding of CAF-mediated immune remodeling is more advanced in HCC than in CCA/iCCA, reflecting differences in the maturity of the respective research fields; accordingly, while this review integrates both entities within a unified framework, the mechanistic depth is necessarily greater for HCC. Importantly, the available evidence in CCA nonetheless supports conserved CAF-driven immunosuppressive programs, although with more limited resolution.

## 2 Methods

Literature was identified through searches of PubMed and Google Scholar using combinations of the terms “hepatocellular carcinoma”, “cholangiocarcinoma”, “cancer-associated fibroblasts”, “tumor microenvironment”, “gut microbiome” and “immune modulation”. This review is based on a comprehensive narrative synthesis of the current literature on CAFs in primary liver cancers. Given the heterogeneity and rapidly evolving nature of the field, a systematic PRISMA-style approach was not applied. Instead, we integrated evidence from key original studies, including recent single-cell and spatial transcriptomic analyses, as well as relevant preclinical and translational reports, to provide a structured conceptual overview of CAF biology and immune regulation in HCC and CCA.

## 3 Liver Immunology

The liver hosts a highly diverse immune compartment, including Kupffer cells, NK/NKT cells, neutrophils, monocytes, and lymphocytes [41]. Its microenvironment is intrinsically tolerogenic, maintaining homeostasis through antigen presentation without strong co-stimulation and IL-10–mediated regulatory T cells (Tregs) expansion [42,43], balanced by anti- and pro-inflammatory cytokines [44].

Chronic liver diseases disrupt immune homeostasis by sustaining inflammatory signaling and promoting chronic inflammation [41]. In chronic HBV infection, persistent exposure to viral antigens induces T-cell exhaustion and apoptosis, weakening immune surveillance [45,46]. HCV instead escapes immune recognition through inhibition of host RNA-sensing pathways and high mutation rates [47,48]. Alcohol abuse and NAFLD further enhance inflammatory signaling and activate innate immune cells such as monocytes, macrophages, and neutrophils [49]. In addition, gut permeability alterations, microbiome dysbiosis, and microbial metabolites contribute to chronic liver disease progression and hepatocarcinogenesis [50,51].

Cholestatic liver diseases are characterized by impaired bile acid metabolism, with accumulation of toxic bile acids promoting hepatocyte injury, DNA damage, inflammation, and cancer development [52]. Bile acids also regulate immune-cell activity, influencing macrophages, Th17, Treg, and NKT cells [53,54]. During cholestatic injury, mast cells, neutrophils, and myeloid-derived suppressor cells contribute to fibrosis, inflammation, and immunosuppression [55,56,57].

In primary biliary cholangitis (PBC), the immune response evolves from an early Th2-dominant profile toward Th1 and later Th17-driven inflammation [58,59,60]. Th17 and MAIT cells produce IL-17, which promotes hepatic stellate cell (HSC) activation and fibrosis [61,62,63,64,65].

Most liver cancers therefore arise in a chronically inflamed and immunosuppressive microenvironment. Increased immune-checkpoint expression and impaired T-cell co-stimulation reduce antitumor immunity, while the TIME supports tumor progression, angiogenesis, immune evasion, and resistance to therapy [66,67,68,69]. In HCC and iCCA, molecular and single-cell studies have revealed distinct TIME subtypes associated with prognosis and response to ICIs [4,70,71,72,73,74].

CAFs are major regulators of the TIME in liver cancers. Through ECM remodeling and secretion of cytokines such as TGF-β, HGF, IGF, and IL-6, CAFs promote tumor proliferation, invasion, angiogenesis, immune escape, and chemoresistance [11,29,75,76,77]. Their marked heterogeneity also suggests the presence of tumor-suppressive CAF subsets, highlighting the complexity and therapeutic relevance of targeting CAF-TIME interactions in liver cancer [26,39,78].

## 4 Origin of CAFs

Hepatic CAFs are a heterogeneous population of activated fibroblasts with diverse origins and functions [29]. Their identification is challenging due to variable expression of common markers, including alpha-smooth muscle actin (α-SMA), fibroblast activation protein (FAP), vimentin, Thy-1 (CD90), and fibroblast-specific protein 1 (FSP-1), none of which are CAF-specific, reflecting their plasticity and complicating lineage tracing [79,80,81,82]. Despite this, most hepatic CAFs in HCC and CCA derive from activated HSCs [29,81]. HSCs acquire a myofibroblast-like phenotype, with increased α-SMA and collagen production, mainly driven by cytokines such as TGF-β [83,84]. Consistently, over 80% of collagen-producing myofibroblasts in experimental liver fibrosis arise from HSCs [85].

Tumor-derived signals further promote HSC-to-CAF transition: HCC cells release exosomal miR-21 and factors such as sulfatase-2, inducing α-SMA^+^/FAP^+^ CAF differentiation, as confirmed by coculture systems and lineage tracing studies identifying HSCs as the main CAF source [79,80,86,87].

In CCA, HSCs are also the predominant CAF source, with <10% derived from portal fibroblasts (PFs) based on scRNA-seq analyses [81]. However, PF contribution varies with disease stage: they prevail in early cholestatic fibrosis, while HSCs dominate in later stages [88]. PFs also contribute to fibrosis in bile duct ligation models and are detected in metastatic iCCA with PF-associated markers [89,90].

Fibrocytes from hematopoietic stem cells can generate CAFs in liver injury via CCR2/CCR1-dependent recruitment [29,91], but appear to play a limited role in CCA, indicating tumor-specific differences [92].

Epithelial-to-mesenchymal transition (EMT) has been proposed as a source of CAFs, with HCC cells and hepatocytes contributing under TGF-β signaling [93,94,95]. However, lineage tracing studies show that hepatocytes and cholangiocytes do not generate myofibroblasts, indicating that EMT-derived CAFs are likely a minor population [96,97].

CAFs can also arise from mesothelial cells, portal fibroblasts, and mesenchymal stromal cells (MSCs) [28]. Mesothelial cells can transdifferentiate into HSCs or myofibroblasts depending on injury context [98,99], while MSCs acquire CAF-like features upon interaction with tumor cells [76,82,100].

Hepatic progenitor cells (HPCs) have recently been identified as an additional source, differentiating into PDGFRα^+^ CAFs under inflammatory conditions and promoting tumor progression [101].

## 5 Activation of CAFs

### 5.1 Tumor–Stroma Crosstalk Is Bidirectional

CAFs promote tumor progression and resistance, while tumor cells and the tumor microenvironment (TME) drive CAF activation, creating a feed-forward loop sustaining ECM remodeling and tumor growth [76]. CAF precursors, including HSCs, are activated via paracrine and exosomal signals.

#### 5.1.1 Growth Factors and Cytokine-Mediated CAF Activation

TGF-β is the central regulator of CAF activation in HCC and CCA. In HCC, TGF-β and CXCL6 induce CAF activation and HSC transdifferentiation [102], while TGF-β promotes CTGF-mediated stromal expansion [103]. In CCA, TGF-β1 drives CAF activation and heparin-binding EGF-like growth factor (HB-EGF) secretion, activating EGFR signaling and reinforcing a feedback loop [104].

Other pathways include platelet-derived growth factor C (PDGF-C)-induced HSC activation [105,106] and TIMP-1-mediated maintenance of the activated fibrogenic phenotype in HCC [107]. In iCCA, tumor-derived PDGF-D promotes CAF recruitment, migration, and lymphangiogenesis via PDGFR-β and Rho GTPases, supporting metastasis [108,109].

Extracellular sulfatase 2 (SULF2) released by HCC cells induces HSC differentiation into CAFs via the TGF-β1/SMAD3 pathway, highlighting convergence of multiple signals on TGF-β–driven activation [79]. ECM components also contribute: hyaluronic acid promotes YAP–CTGF signaling, sustaining CAF activation [110,111], while the Sox9/INHBB axis and periostin further enhance HSC activation through Smad and integrin–FAK–STAT3 pathways [112,113].

#### 5.1.2 Senescence and Exosome-Mediated CAF Activation

Senescence pathways also regulate CAFs. In HCC, CAFs can acquire a senescence-associated secretory phenotype (SASP), with increased pro-inflammatory and tumor-promoting factors [114]. BMP4 further enhances this program [115].

Tumor-derived exosomes contribute to CAF activation by transferring oncogenic non-coding RNAs that modulate stromal cell behavior. In particular, exosomal miR-1247-3p promotes fibroblast activation at metastatic sites via β1-integrin–NF-κB signaling [87,116,117].

#### 5.1.3 Non-Coding RNAs and CAF Activation

Circular RNAs (circRNAs) are a class of non-coding RNAs characterized by covalently closed continuous loops. Dysregulated circRNA expression has been implicated in the development and progression of several cancers, including HCC and CCA [118,119]. It has been reported that circ_0020256 stimulates CAF activation via the EIF4A3/KLF4 pathway and is increased in CCA. Increased circ_0020256 induces CCA cells to secrete TGF-β1, which activates CAFs by phosphorylating Smad2/3. The negative effects of circ_0020256 silencing on TGF-β1/Smad2/3-mediated CAF activation are reversed by KLF4 overexpression. Moreover, by inhibiting autophagy, CAF-derived IL-6 further enhances CCA cell proliferation, migration, and EMT, supporting their protumorigenic role [120].

#### 5.1.4 Alternative CAF Precursors and Mechanical Regulation

Beyond HSC-derived CAFs, portal fibroblasts (PFs) can be activated by tumor signals such as lysophosphatidic acid [121]. In CCA, mesothelin (MSLN) enhances PF activation by promoting TGF-β1 signaling through disruption of the THY-1–TGFβR1 complex, driving fibrogenesis [92,122,123,124].

Mechanical cues also regulate CAF activation: matrix stiffness activates HSCs via CD36–AKT–E2F3 and RHOA–AKT pathways, enhancing fibroblast activation and tumor progression, with YAP1^+^ CAFs reinforcing this feedback loop [125,126,127]. Additionally, placental growth factor (PlGF) promotes desmoplasia in iCCA, and its inhibition reduces myofibroblast activation and tissue stiffness [128].

#### 5.1.5 Metabolic and Post-Translational Regulation of CAFs

Emerging evidence indicates that CAF activation in CCA may also be linked to tumor metabolic cues, such as bile acid overload. Exposure to high levels of bile acids stimulates G protein–coupled bile acid receptor 1 (GPBAR1) signaling on CAFs, inducing CXCL10 expression, which enhances EMT, tumor invasion, and neutrophil recruitment, thereby fostering an immunosuppressive TIME [129].

NEDDylation, a post-translational protein modification, has recently been implicated in CAF regulation. Reduced NEDDylation in CCA cells disrupts the secretion of CAF-activating factors, angiogenic mediators, and oncogenic signals, thereby impairing CAF proliferation and migration [130], suggesting that NEDDylation may represent an upstream regulator of fibroblast activation. Factors implicated in CAF activation are summarized in Table 1. 

**Table 1:** Cancer associated fibroblasts (CAFs) activation factors in liver cancer.

Tumor Type	Activation Factors	Function	References
**Hepatocellular carcinoma (HCC)**	Transforming growth factor beta (TGF-β) and C-X-C motif chemokine ligand 6 (CXCL6)	Canonical profibrotic cytokine and inflammatory chemokine that promote HSCs/CAF activation, ECM remodeling and tumor progression.	[102]
	Connective tissue growth factor (CTGF)	Matricellular protein induced by TGF-β that enhances fibroblast activation, ECM deposition and desmoplasia.	[103]
	PDGF-C	Growth factor stimulating fibroblast proliferation, activation and matrix production.	[105,106]
	TIMP-1	Regulates matrix remodeling while promoting fibroblast survival and CAF activation.	[107]
	SULF2	Extracellular endosulfatase that remodels heparan sulfate proteoglycans, enhancing growth factor signaling involved in CAF activation.	[79]
	HA	Major ECM glycosaminoglycan that promotes fibroblast activation through mechanotransduction and CD44 signaling.	[110]
	Activin B	TGF-β superfamily ligand that induces myofibroblastic differentiation and ECM production.	[113]
	Periostin	Matricellular ECM protein that supports CAF activation, collagen organization and tumor invasion.	[112]
	Bone morphogenetic protein 4 (BMP4)	TGF-β family cytokine promoting fibroblast activation and acquisition of a protumoral phenotype.	[131]
	miRNA-21	Exosomal microRNA that reprograms fibroblasts toward a CAF phenotype by regulating profibrotic signaling pathways.	[87]
	miR-1247-3p	Tumor-derived exosomal miRNA that activates fibroblasts through inflammatory signaling (e.g., β1-integrin/NF-κB pathway).	[117]
	LPA	Bioactive phospholipid that stimulates fibroblast migration, contractility and activation.	[121]
	Matrix stiffness	Increased ECM rigidity activates fibroblasts via mechanosensitive pathways (YAP/TAZ, integrins, FAK), reinforcing desmoplasia.	[125]
**Intrahepatic cholangiocarcinoma (iCCA)**	Placental growth factor (PlGF)	VEGF family member that activates CAFs and promotes stromal remodeling, angiogenesis and tumor progression.	[128]
	Platelet-derived growth factor D (PDGF-D)	Activator of fibroblasts driving CAF differentiation, ECM remodeling and lymphangiogenic signaling.	[108,109]
**CCA**	TGFβ	Central profibrotic cytokine promoting CAF activation and deposition of desmoplastic ECM.	[104]
	circ_0020256	Tumor-derived circular RNA involved in fibroblast reprogramming toward a CAF phenotype through intercellular communication.	[120]
	Mesothelin (MSLN)	Cell-surface glycoprotein that enhances CAF activation and tumor–stroma interactions.	[122,123]
	Bile acids	Accumulated bile acids stimulate fibroblast activation through inflammatory and profibrotic signaling pathways.	[129]
	NEDDylation	Post-translational modification pathway whose activation promotes CAF differentiation and maintenance of the protumoral stromal phenotype.	[130]

Note: BMP4, bone morphogenetic protein 4; CAF, cancer-associated fibroblast; CTGF, connective tissue growth factor; CXCL6, C-X-C motif chemokine ligand 6; ECM, extracellular matrix; HA, hyaluronic acid; HCC, hepatocellular carcinoma; HSC, hepatic stellate cell; iCCA, intrahepatic cholangiocarcinoma; LPA, lysophosphatidic acid; miR/miRNA, microRNA; MSLN, mesothelin; NEDDylation, NEDD8-mediated post-translational modification; NF-κB, nuclear factor kappa B; PDGF-C, platelet-derived growth factor C; PDGF-D, platelet-derived growth factor D; PlGF, placental growth factor; SULF2, sulfatase 2; TGF-β, transforming growth factor beta; TIMP-1, tissue inhibitor of metalloproteinases 1; YAP/TAZ, Yes-associated protein/transcriptional coactivator with PDZ-binding motif.

## 6 CAF Heterogeneity and Functional Classification in HCC and CCA

scRNA-seq and spatial transcriptomics reveal that CAFs in liver cancer are highly heterogeneous and functionally specialized rather than a uniform population [29,34,75,132]. They can be classified into distinct transcriptional and spatial subtypes with specific roles in ECM remodeling, tumor progression, and immune regulation [133,134]. This heterogeneity is key to understanding how CAFs shape the TIME and influence therapeutic responses [76,133,135,136]. Selected CAF populations with well-defined phenotypic, functional, and spatial features are summarized in Table 2.

### 6.1 CAF Subtypes in HCC

In HCC, CAF heterogeneity is organized along transcriptional, spatial, and functional axes, with distinct subsets occupying specific niches and contributing to tumor structure, immune modulation, and progression [75]. This complexity is further increased by the cirrhotic background.

Single-cell studies identify multiple CAF subtypes, including myofibroblastic CAFs (myCAFs), inflammatory CAFs (iCAFs), vascular CAFs (vCAFs), cytokine- and growth factor-expressing CAFs (cyCAFs), antigen-presenting CAFs (apCAFs), proliferative CAFs (pCAFs), lipid-processing myCAFs (lpmCAFs), and lipid metabolism and processing CAFs (lpCAFs) [102,132], although their nomenclature and classification vary across datasets.

Spatial analyses also reveal immunomodulatory CAFs, such as IL34^+^ CAFs influencing immunotherapy response, and FAP^+^ or PDGFRA^+^ populations whose localization correlates with clinical outcomes [137,138].

Integrative single-nucleus and spatial transcriptomics have identified hypoxic metabolic myofibroblasts (hmmyCAFs), a myCAF subset localized at the invasive front of HCC, where they form collagen-rich barriers that exclude CD8^+^ T cells and promote an immune-excluded phenotype [139]. 

Metabolically specialized CAFs, such as CD36^+^ subsets involved in lipid metabolism and immunosuppression, further highlight the link between stromal metabolism and immune dysfunction in HCC [40].

Not all CAF subsets are tumor-promoting. FMO2^+^ CAFs are associated with better prognosis and improved response to anti-PD-1 therapy, promoting tertiary lymphoid structures and enhancing CD8^+^ T-cell and M1 macrophage infiltration via CCL19–CCR7 signaling [140]. This CAF subset has been identified in single-cohort analyses and requires independent validation across larger datasets.

CAF heterogeneity in HCC is closely linked to spatial localization. Distinct subsets occupy specific niches, such as HLA-DRB1^+^ CAFs in non-tumoral areas, MMP11^+^ at tumor borders, and VEGFA^+^ CAFs in hypoxic cores, the latter associated with poor outcomes and representing a terminal, hypoxia-driven state [139,141,142].

Importantly, CAFs can exert context-dependent and even opposing functions in HCC. While several CAF subsets promote tumor progression and immune evasion, specific myofibroblastic or collagen-producing CAF populations have also been reported to exert tumor-restraining effects. These observations do not necessarily conflict with the pro-tumoral role of hmmyCAFs, which represent a distinct hypoxia-associated myofibroblastic state characterized by metabolic reprogramming, collagen-rich barrier formation, and CD8^+^ T-cell exclusion. Single-cell and proteomic studies therefore highlight substantial functional heterogeneity even among phenotypically related CAF populations, underscoring the importance of cellular state and spatial context in determining CAF function [76,86,138,143].

### 6.2 CAF Subtypes in iCCA

In CCA, scRNA-seq reveals marked CAF heterogeneity, with multiple subclusters differing in transcriptional profiles, origin, and function [81,144,145]. The largest population is vCAFs, expressing microvascular markers and inflammatory chemokines, and involved in hypoxia response and stromal remodeling [144]. Matrix CAFs (mCAFs) instead express ECM-related genes and contribute to collagen organization and tissue structure [144,145].

Other subsets include iCAFs, which combine ECM remodeling with immune signaling [39,81,144,145], and apCAFs, expressing MHC class II molecules and participating in adaptive immune regulation [144,145]. Additional populations include EMT-like CAFs (eCAFs) and lipofibroblast-likeCAFs, reflecting further functional diversity within the CCA stroma [144]. 

Spatial organization further defines CAF function in CCA. CD146^+^ vCAFs localize to tumor cores and vascular regions, supporting proliferation and angiogenesis, while POSTN^+^ mCAFs are enriched at invasive fronts, associating with ECM remodeling and invasion [144,146]. These spatial and transcriptional programs are consistent across cohorts, including ICI-treated tumors, highlighting their clinical relevance [144,147]. 

CAF-derived mediators also have context-dependent roles. For instance, COL1A1 contributes to ECM stiffness but does not directly promote tumor growth in iCCA [81,86].

**Table 2:** Functional classification of CAF subtypes in HCC and iCCA.

Tumor Type	CAF Subtype	Key Markers	Main Functions	Spatial Localization	Immunological Role
**HCC**	myCAFs	ACTA2 (α-SMA), COL1A1	ECM production, fibrosis	Fibrotic stroma	Can restrict or promote tumor growth
	iCAFs	IL6, CXCL chemokines	Inflammatory signaling	Stromal regions	Immunosuppressive cytokine production
	vCAFs	VEGFA	Angiogenesis	Hypoxic tumor core	Associated with poor prognosis
	apCAFs	MHC-II genes	Antigen presentation	Variable	T-cell modulation
	hmmyCAFs	POSTN	ECM barrier, hypoxia adaptation	Invasive front	CD8^+^ T-cell exclusion, ICI resistance
	CD36^+^ CAFs	CD36	Lipid metabolism	Tumor core	T-cell dysfunction
	FMO2^+^ CAFs	FMO2	TLS formation	Immune niches	Enhance anti-tumor immunity
	IL34^+^ CAFs	IL34	Immune modulation	Tumor stroma	Influence ICI response
**iCCA**	vCAFs	MCAM (CD146), MYH11, RGS5	Vascular remodeling, angiogenesis	Tumor core, perivascular regions	Promote immune cell recruitment and inflammation
	mCAFs	COL5A1, FN1, POSTN	ECM deposition, tissue stiffness	Invasive front	Physical barrier, immune exclusion
	iCAFs	CXCL1, SAA1, C3	Inflammation, ECM remodeling	Diffuse stromal areas	Cytokine-mediated immune modulation
	apCAFs	CD74, HLA-DRA	Antigen presentation	Stromal niches	Modulate adaptive immunity
	eCAFs	KRT8, KRT19	EMT-like features	Tumor–stroma interface	Function unclear/context-dependent
	Lipofibroblast-like	APOA2, FABP1	Lipid metabolism	Adjacent liver tissue	Metabolic modulation

Note: ACTA2, actin alpha 2, smooth muscle; α-SMA, alpha-smooth muscle actin; APOA2, apolipoprotein A2; apCAFs, antigen-presenting cancer-associated fibroblasts; C3, complement component 3; CAFs, cancer-associated fibroblasts; CD36, cluster of differentiation 36; CD74, cluster of differentiation 74; COL1A1, collagen type I alpha 1 chain; COL5A1, collagen type V alpha 1 chain; CXCL, C-X-C motif chemokine ligand; CXCL1, C-X-C motif chemokine ligand 1; eCAFs, epithelial-like cancer-associated fibroblasts; ECM, extracellular matrix; EMT, epithelial-to-mesenchymal transition; FABP1, fatty acid-binding protein 1; FMO2, flavin-containing dimethylaniline monooxygenase 2; FMO2^+^ CAFs, FMO2-positive cancer-associated fibroblasts; FN1, fibronectin 1; HCC, hepatocellular carcinoma; HLA-DRA, major histocompatibility complex class II, DR alpha; hmmyCAFs, hypoxic metabolic myofibroblasts; iCAFs, inflammatory cancer-associated fibroblasts; iCCA, intrahepatic cholangiocarcinoma; ICI, immune checkpoint inhibitor; IL6, interleukin 6; IL34, interleukin 34; IL34^+^ CAFs, IL34-positive cancer-associated fibroblasts; KRT8, keratin 8; KRT19, keratin 19; MCAM, melanoma cell adhesion molecule; MHC-II, major histocompatibility complex class II; mCAFs, matrix-producing cancer-associated fibroblasts; myCAFs, myofibroblastic cancer-associated fibroblasts; MYH11, myosin heavy chain 11; POSTN, periostin; RGS5, regulator of G protein signaling 5; SAA1, serum amyloid A1; TLS, tertiary lymphoid structure; vCAFs, vascular cancer-associated fibroblasts; VEGFA, vascular endothelial growth factor A.

Collectively, these findings demonstrate that CAF heterogeneity in both HCC and CCA is defined by integrated transcriptional, spatial, and functional programs. These spatial transcriptomic observations are based on limited patient cohorts and should therefore be interpreted as descriptive and hypothesis-generating. Distinct CAF subsets exert specialized roles in ECM remodeling, vascular regulation, immune cell recruitment, and metabolic adaptation, thereby actively shaping the TIME and influencing therapeutic responses [29,40,81,148]. This diversity reflects functionally specialized stromal states—including myofibroblastic, inflammatory, vascular, matrix, and antigen-presenting CAFs—whose roles extend beyond lineage and include active immune regulation through cytokine production, antigen presentation, and modulation of immune cell trafficking [29,40,81,149].

The functional specialization of CAFs naturally positions them as central modulators of the liver TIME, influencing the recruitment, activation, and suppression of resident and infiltrating immune cells. Accordingly, understanding CAF-immune cell interactions emerge as a critical step for dissecting mechanisms of immunosuppression, chronic inflammation, and therapeutic response in primary liver cancers [29,40,148]. In addition, the interpretation of single-cell and spatial transcriptomic datasets increasingly relies on advanced computational and artificial intelligence-assisted approaches, including machine learning-based clustering, trajectory inference, cell–cell communication modeling, spatial mapping algorithms, and integrative multi-omics frameworks [150], which are becoming essential for resolving CAF heterogeneity, reconstructing stromal–immune interactions, and identifying clinically relevant cellular states within liver tumors.

Fig. 1 provides a schematic overview of the different cellular origins of CAFs, the main signaling pathways involved in their activation, and the functional heterogeneity of CAF subpopulations in HCC and iCCA.

**Figure 1:**
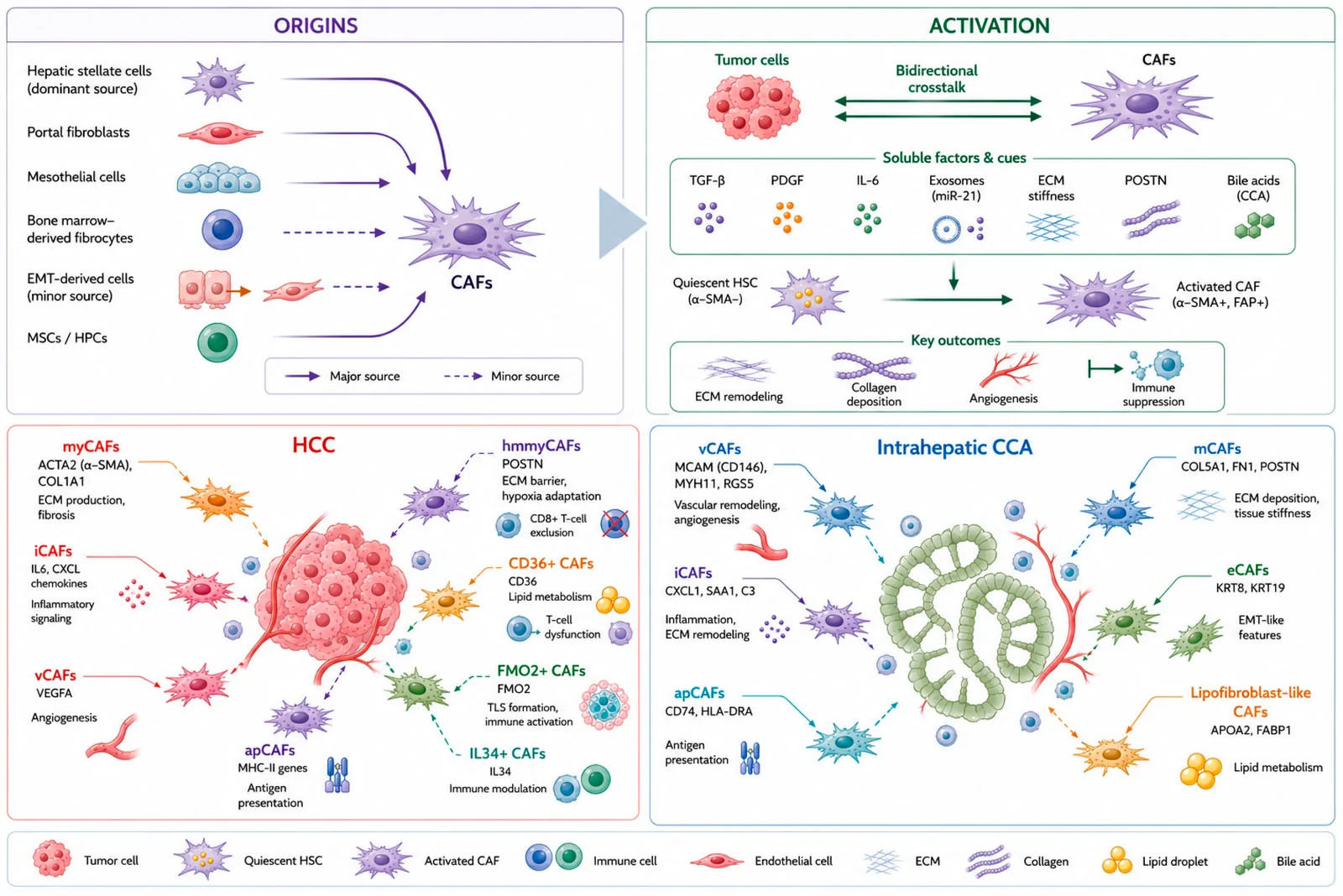
Origins, activation, and functional heterogeneity of cancer-associated fibroblasts in primary liver cancers. Cancer-associated fibroblasts (CAFs) originate predominantly from hepatic stellate cells (HSCs), but also from portal fibroblasts (PFs), mesothelial cells, fibrocytes, mesenchymal stem cells/hepatic progenitor cells (MSCs/HPCs), and, to a lesser extent, cells undergoing epithelial-to-mesenchymal transition (EMT). Tumor-derived factors and microenvironmental cues, including transforming growth factor beta (TGF-β), platelet-derived growth factor (PDGF), interleukin-6 (IL-6), exosomal microRNAs (miRNAs), extracellular matrix (ECM) stiffness, periostin (POSTN), and bile acids, promote CAF activation and transdifferentiation, leading to ECM remodeling, angiogenesis, fibrosis, and immune suppression. Distinct CAF subtypes with specialized functions are observed in hepatocellular carcinoma (HCC) and intrahepatic cholangiocarcinoma (iCCA), highlighting the transcriptional and spatial heterogeneity of the liver tumor stroma. Created with AI-assisted illustration tools.

### 6.3 Emerging Experimental Models to Study CAF Heterogeneity and Immune Interactions

The identification of transcriptionally and functionally distinct CAF subsets has created an urgent need for experimental models capable of functionally validating their biological roles and interactions within the TME. While single-cell and spatial transcriptomics have greatly expanded our understanding of CAF diversity and stromal–immune interactions, conventional two-dimensional cultures remain insufficient to recapitulate the architectural, mechanical, and cellular complexity of the liver TME [151,152].

In particular, co-culture systems combining patient-derived organoids (PDOs) with cancer-associated fibroblasts have demonstrated that CAFs provide a supportive stromal niche that promotes tumor growth and therapy resistance, highlighting the critical contribution of fibroblast–tumor interactions to disease progression [78].

The incorporation of additional stromal components has further improved the physiological relevance of these models. In HCC, organoid co-cultures containing stromal and endothelial cells have been shown to recapitulate angiocrine signaling and generate inflammatory TME, providing a valuable platform to investigate multicellular crosstalk, cytokine networks, and tumor progression mechanisms [153].

Similarly, the integration of effector T cells into CCA PDOs enables the investigation of antitumor immune responses, immune evasion mechanisms, and patient-specific sensitivity to immunotherapeutic strategies [154].

More recently, microfluidic tumor-on-chip technologies have enabled the reconstruction of multicellular CCA microenvironments incorporating tumor cells, CAFs, endothelial cells, and immune populations within spatially organized three-dimensional structures. These systems allow the dynamic study of stromal remodeling, angiogenesis, immune-cell trafficking, and therapeutic responses under conditions that more closely mimic *in vivo* tumor architecture [155].

Collectively, organoid co-cultures, scaffold-based systems, and microfluidic platforms represent promising translational tools for bridging the gap between descriptive stromal profiling and mechanistic understanding. By enabling the functional interrogation of specific CAF subsets and their interactions with immune populations, these models may facilitate biomarker discovery, patient stratification, drug testing, and the development of personalized stromal-targeted and immunomodulatory therapies in primary liver cancers.

### 6.4 Metabolic Functions of CAFs in the Liver TME

CAFs contribute to the metabolic reprogramming of tumor cells in ways that profoundly influence the immune landscape of liver cancers [40,156,157]. In HCC, specific CAF subsets such as CD36^+^ CAFs promote lipid metabolic rewiring by enhancing fatty acid uptake and utilization, thereby supporting tumor proliferation, migration, recurrence and metastasis [40,156,158]. CAFs also engage glycolytic pathways and can increase glucose consumption and lactate release, contributing to a tumor-supportive metabolic shift [156,157,159,160]. Through secretion of cytokines and growth factors, including IL-6, TGF-β and HGF, CAFs further drive epithelial–mesenchymal transition, metabolic reprogramming, stemness and angiogenesis, reinforcing tumor progression and resistance to therapy [40,156,158,161,162]. 

The resulting accumulation of lactate and other immunomodulatory metabolites leads to acidification of the TME and nutrient competition, thereby promoting T-cell dysfunction and exhaustion, impairing the cytotoxic activity, proliferation, and cytokine production of CD8^+^ T cells and NK cells, while favoring the expansion of Tregs, M2-like macrophages, and myeloid-derived suppressor cells (MDSCs) [11,156,159,163]. In HCC, CD36^+^ CAFs and POSTN^+^ CAFs exemplify how metabolically and cytokine active CAF subsets recruit and polarize suppressive myeloid populations (e.g., CD33^+^ MDSCs, SPP1^+^ macrophages) via migration inhibitory factor (MIF)–CD74 and IL 6/STAT3 signaling, thereby limiting responses to immunotherapy [40,148,158]. 

In CCA, CAF-mediated metabolic remodeling also involves amino acid metabolism and 5 lipoxygenase–dependent pathways in CD33^+^ MDSCs, with CAF derived IL 6 and IL 33 promoting leukotriene B4 production and tumor stemness, again coupling metabolic reprogramming to immune suppression [76,164].

Collectively, CAF-driven metabolic reprogramming emerges as both a mechanism of tumor adaptation and a critical determinant of immunosuppression and therapeutic resistance in primary liver cancers [29,132,163].

### 6.5 Gut–Liver Axis as a Regulator of CAF Biology

Increasing evidence supports a critical role for the gut–liver axis in the development and progression of primary liver cancers, including HCC and CCA [165]. Dysbiosis and increased intestinal permeability facilitate the translocation of microbial products, such as lipopolysaccharide (LPS) and other pathogen associated molecular patterns (PAMPs), which activate inflammatory and immune signaling pathways in the liver and biliary tract, thereby promoting chronic inflammation, fibrosis, and tumorigenesis [165,166,167,168].

Beyond their direct effects on epithelial and immune cells, microbiota derived signals are important modulators of the stromal compartment. In chronic liver disease and HCC, dysbiosis and gut derived microbial/metabolic products (such as LPS and secondary bile acids like deoxycholic acid) are linked to persistent hepatic inflammation, activation and senescence of HSCs, ECM deposition and fibrosis, contributing to a tumor promoting microenvironment [169,170]. Since activated HSCs are a major source of myofibroblasts/CAF like cells and key producers of ECM in HCC, this supports the idea that microbiota driven inflammatory pathways can indirectly shape CAF biology and stromal evolution during tumor progression [38,171].

Although direct evidence linking the gut microbiome to CAF regulation in CCA is still lacking, CCA is characterized by a pronounced desmoplastic stroma rich in fibroblasts and ECM, and CAFs are central to matrix remodeling, angiogenesis, and tumor–stroma crosstalk in liver cancer [38,171]. These observations support the plausibility of a microbiota–stroma axis also in CCA, but dedicated mechanistic studies are needed to clarify how gut microbial communities influence stromal cell populations and their interactions with tumor and immune cells in primary liver cancers [172].

## 7 Immunomodulatory Roles of CAFs in HCC and CCA

CAFs play a central role in orchestrating immune regulation within the TIME [102]. Rather than acting as direct immune effectors, CAFs shape immune cell recruitment, spatial distribution, and functional states through ECM remodeling, secretion of cytokines and chemokines, and direct crosstalk with immune populations. In primary liver cancers, CAF-driven immune remodeling contributes to immune exclusion, suppression of cytotoxic responses, and resistance to immunotherapy, establishing CAFs as key stromal regulators of antitumor immunity. Nevertheless, current evidence on CAF–immune interactions in CCA, is primarily based on small-scale transcriptomic and histopathological studies and remains largely correlative in nature.

### 7.1 CAF-Centered Networks Shaping Lymphocyte Function in HCC

Across recent mechanistic studies, a consistent picture is emerging in liver cancer in which specific CAF states actively program lymphocyte exclusion and dysfunction through complementary routes, rather than simply providing structural support.

#### 7.1.1 CAF-Mediated T-Cell Exclusion and Impaired Recruitment

POSTN^+^ CAFs represent a key stromal subset implicated in immune exclusion through ECM remodeling (see Section 6.1 for detailed mechanisms). In HCC, Wang et al. integrated large-scale scRNA-seq with spatial transcriptomics and multiplex immunofluorescence to define POSTN^+^ CAFs as a myofibroblastic population enriched at tumor borders, where they form a stromal “immune barrier” that restricts CD8^+^ T-cell infiltration and impairs responsiveness to ICB [148]. Mechanistically, POSTN^+^ CAFs promote ECM remodeling and engage in reciprocal crosstalk with SPP1^+^ macrophages via IL-6/STAT3 signaling, reinforcing an immunosuppressive microenvironment [148]. Using complementary single-cell and spatial transcriptomic approaches, Chen et al. demonstrated that POSTN^+^ CAF–rich stromal regions are associated with reduced T-cell infiltration and enrichment of exhausted CD8^+^ T cells, providing spatial evidence that POSTN^+^ CAFs actively shape lymphocyte localization and functional state [173]. Together, these studies establish POSTN^+^ CAFs as central stromal drivers of lymphocyte exclusion and immune suppression, suggesting their relevance as potential targets to enhance antitumor immunity and supporting the idea that CAF composition can be a determinant of immunotherapy outcome [148,173].

A conceptually aligned but mechanistically distinct axis is described for endosialin (CD248)^+^ CAFs, where immune exclusion is driven less by physical remodeling and more by chemokine suppression: endosialin restrains STAT1 phosphorylation/nuclear translocation in CAFs, leading to decreased CXCL9/10 output and consequently reduced CD8^+^ T-cell recruitment, with high endosialin expression in patient tumors correlating with low CD8^+^ infiltration [174]. Importantly, endosialin targeting synergizes with anti-PD-1, consistent with the idea that restoring trafficking can convert non-inflamed tumors (the so called “cold” HCC) into immunotherapy-responsive ones [174].

#### 7.1.2 CAF-Driven T-Cell Exhaustion and Senescence

By integrating transcriptomic profiling, immune deconvolution, spatial transcriptomics, and histopathological analyses, Murai et al. [175] identified a subset of steatotic, nonviral HCC characterized by an immune-enriched yet immune-exhausted TME. Spatial transcriptomic mapping demonstrated that exhausted CD8^+^ T cells preferentially localize within CAF-rich stromal regions with active TGF-β signaling, in close proximity to M2 macrophages. These findings provide direct spatial evidence that CAFs participate in the formation of immunosuppressive niches that promote T-cell exhaustion rather than simple immune exclusion, thereby linking metabolic alterations such as intratumoral steatosis to CAF–lymphocyte crosstalk and immunotherapy responsiveness in HCC [175].

In line with the concept that CAFs regulate T-cell functional fitness rather than simply controlling the extent of lymphocyte infiltration, Zhang and colleagues identified a distinct subgroup of CAFs characterized by the expression of CD90 and laminin subunit α4 (LAMA4) that actively imposes a senescence program on CD8^+^ T cells [176]. These CD90^+^ LAMA4^+^ ECM CAFs spatially co-localize with CD8^+^ T cells in liver tumors, indicating that T-cell recruitment is not prevented. Instead, CAF-derived LAMA4 seems to engage the ITGA6 receptor on CD8^+^ T cells, triggering a DNA damage response associated with increased expression of the senescence markers p16 and p21, together with activation of p53 signaling, ultimately resulting in loss of proliferative and cytolytic capacity. Importantly, this CAF-driven T-cell dysfunction is mechanistically distinct from canonical exhaustion, as it reflects an irreversible senescent state rather than a checkpoint-mediated inhibitory program. In fact, the authors showed that blocking LAMA4 limits malignant progression and improves response to anti-PD-1 *in vivo*, providing another rationale for why T-cell–infiltrated HCCs may remain refractory to ICB [176].

#### 7.1.3 FAP^+^ CAFs and Immune-Checkpoint Activation

As indicated in studies regarding several cancer types, the high expression of FAP in CAFs is associated with an increasing infiltration of Tregs and M2 macrophages and a decreasing infiltration of CD8^+^ T cells and NK cells in the TME [177]. In this regard, a study on FAP in HCC supports the clinical relevance of an “activated CAF–immune checkpoint” state: high FAP associates with poorer prognosis and correlates with immune infiltration patterns and higher expression of PD-1/CTLA-4 and PD-L1/PD-L2, suggesting that CAF-rich tumors may couple stromal activation to compensatory checkpoint pathways that further restrain lymphocyte function [178].

Taken together, these studies delineate three nonredundant CAF-driven immune-evasion modes in HCC: (i) T-cell exclusion mediated by barrier-like stromal niches reinforced by myeloid crosstalk (POSTN^+^ CAFs interacting with SPP1^+^ macrophages) [148,173]; (ii) impaired T-cell trafficking due to active chemokine suppression (endosialin^+^ CAF–mediated inhibition of CXCL9/10) [174]; and (iii) T-cell dysfunction despite infiltration, enforced through CAF-induced exhaustion [175] or senescence programs (CD90^+^ LAMA4^+^ CAFs) [176]. While mechanistically compelling, these findings are based on limited patient material and experimental models and require further validation in larger clinical cohorts. These mechanisms, together with CAF-rich TGF-β–active and FAP^+^ stromal states associated with immune exhaustion and immune checkpoint upregulation [175,177,178], converge on the concept that CAF heterogeneity underlies layered immune suppression in liver cancer, providing a strong rationale for CAF-subtype–informed combinatorial strategies integrating stromal targeting with PD-1 blockade to achieve durable lymphocyte-mediated tumor control (see Table 3).

### 7.2 CAF-Centered Networks Shaping Lymphocyte Function in CCA

In CCA, the lymphoid infiltrate is profoundly shaped by the highly desmoplastic stroma, positioning CAFs as central regulators of both lymphocyte composition and functional state rather than passive bystanders [179]. 

#### 7.2.1 CAF-Mediated Spatial Organization of T-Cell Responses

Early immunohistochemical and systematic analyses revealed that CCA is generally characterised by poor intratumoural infiltration of cytotoxic CD8^+^ T cells and B cells, with preferential localisation in peritumoural regions, alongside enrichment of immunosuppressive FoxP3^+^ regulatory T cells within the tumour core, a spatial pattern associated with adverse prognosis [180].

Mechanistic insight into CAF–lymphocyte crosstalk in CCA emerged from studies linking CAF senescence states to immune modulation, showing that Caveolin-1 (CAV1)-expressing CAFs correlate with increased FoxP3^+^ T-cell infiltration and poor survival, independently of CD8^+^ T-cell density, suggesting that CAFs selectively promote regulatory rather than effector lymphoid compartments [181].

#### 7.2.2 CAF-Mediated Suppression of B-Cell Immunity

More recent high-resolution analyses have expanded this concept beyond T cells, demonstrating that CAFs actively suppress B-cell infiltration, maturation, and effector function. In intrahepatic CCA, tumour-infiltrating B cells are scarce, immature, and functionally impaired, whereas peritumoural regions harbour mature tertiary lymphoid structures associated with favourable outcomes; importantly, direct co-culture with CAFs or CAF-derived soluble factors was sufficient to inhibit B-cell differentiation and activation, identifying CAFs as dominant stromal checkpoints of humoral immunity [182]. These immunosuppressive effects are mediated, at least in part, by CAF-derived IL-6 and TGF-β, which emerge as convergent pathways driving lymphocyte dysfunction across different immune lineages in CCA [182].

Collectively, these findings, although still limited, seem to support a model in which CAFs in CCA orchestrate a selective and spatially organised lymphoid exclusion and reprogramming, favouring regulatory and dysfunctional lymphocyte states while limiting effective antitumour immunity, thereby contributing to immune escape and limited responsiveness to immunotherapy (see Table 3).

**Table 3:** CAFs and lymphocytes crosstalk in liver cancers.

Tumor Type	CAF Subtype/Marker	Main CAF Function	Effect on Lymphocytes	Dominant Lymphocyte Phenotype	Key Molecular Mechanisms	Clinical/Therapeutic Implications	Ref
**HCC**	POSTN^+^ CAFs	Stromal immune barrier formation	Reduced CD8^+^ T-cell infiltration	Immune exclusion; exhausted CD8^+^ T cells	ECM remodeling; POSTN–integrin signaling; IL-6/STAT3-mediated crosstalk with SPP1^+^ macrophages	Poor response to ICI; POSTN^+^ CAF abundance predicts immunotherapy resistance	[148,173]
	POSTN^+^ CAFs + SPP1^+^ macrophages	Myeloid–stromal immune reinforcement	Amplified immune exclusion	Exhausted CD8^+^ T cells	Reciprocal IL-6/STAT3 signaling; osteopontin-driven macrophage recruitment	Defines immunosuppressive niches; combinatorial stromal–myeloid targeting	[148,173]
	Endosialin (CD248)^+^ CAFs	Chemokine suppression	Impaired CD8^+^ T-cell recruitment	Immune-cold phenotype	STAT1 inhibition → ↓ CXCL9/10	Endosialin blockade synergizes with PD-1 inhibition	[174]
	CAF-rich TGF-β–active stroma (steatotic HCC)	Immunosuppressive niche formation	T cells present but dysfunctional	Exhausted CD8^+^ T cells	TGF-β signaling; proximity to M2 macrophages	Explains ICI resistance in immune-infiltrated nonviral HCC	[175]
	CD90^+^ LAMA4^+^ eCAFs	Active T-cell functional reprogramming	CD8^+^ T cells present but inactivated	Senescent CD8^+^ T cells (non-exhausted)	LAMA4–ITGA6 signaling → DNA damage response (p16, p21, p53)	LAMA4 targeting restores ICI sensitivity	[176]
	FAP^+^ CAFs	Global immune checkpoint coupling	↓ CD8^+^ T cells and NK cells; ↑ Tregs and M2 macrophages	Checkpoint-high, ineffective T cells	Association with PD-1, CTLA-4, PD-L1/PD-L2 expression	Poor prognosis; CAF–checkpoint axis	[177,178]
**CCA**	CAF-rich desmoplastic stroma	Spatial immune restriction	Low intratumoural CD8^+^ T and B cells	Immune-excluded phenotype	Dense ECM; stromal sequestration	Baseline immune-cold TME	[179,180]
	CAV1^+^ senescent CAFs	Selective immune skewing	↑ FoxP3^+^ Tregs without affecting CD8^+^ density	Regulatory T-cell enrichment	CAF senescence; IL-6-linked signaling	Poor OS; CAF senescence as prognostic marker	[181]
	CAFs (IL-6/TGF-β-producing)	Humoral immune suppression	Impaired B-cell differentiation and activation	Immature, dysfunctional B cells	IL-6 and TGF-β signaling	Reversible with cytokine blockade	[182]
	CAF-driven B-cell exclusion	TLS restriction to peritumoural areas	↓ intratumoural B cells; preserved peritumoural TLS	Loss of effector humoral immunity	CAF–B cell soluble factor crosstalk	TLS presence predicts better prognosis	[182]

Note: CAF, cancer-associated fibroblast; CAV1, caveolin 1; CD8, cluster of differentiation 8; CD90, cluster of differentiation 90; CD248, cluster of differentiation 248; CCA, cholangiocarcinoma; CTLA-4, cytotoxic T-lymphocyte-associated protein 4; CXCL9/10, C-X-C motif chemokine ligands 9 and 10; ECM, extracellular matrix; eCAFs, extracellular matrix-producing cancer-associated fibroblasts; FAP, fibroblast activation protein; FoxP3, forkhead box P3; HCC, hepatocellular carcinoma; ICI, immune checkpoint inhibitor; IL-6, interleukin 6; ITGA6, integrin subunit alpha 6; LAMA4, laminin subunit alpha 4; M2 macrophages, alternatively activated macrophages; NK cells, natural killer cells; OS, overall survival; PD-1, programmed cell death protein 1; PD-L1, programmed death-ligand 1; PD-L2, programmed death-ligand 2; POSTN, periostin; SPP1, secreted phosphoprotein 1; STAT1, signal transducer and activator of transcription 1; STAT3, signal transducer and activator of transcription 3; TGF-β, transforming growth factor beta; TLS, tertiary lymphoid structure; TME, tumor microenvironment; Tregs, regulatory T cells.

### 7.3 CAF–Macrophages Crosstalk in HCC

In HCC, as in other solid malignancies, CAFs and tumor-associated macrophages (TAMs) form a tightly interconnected stromal network that fuels tumor progression, immune escape, and therapeutic resistance [183]. Largely derived from activated HSCs, CAFs orchestrate macrophage recruitment and polarization through paracrine mediators such as CXCL12, TGF-β, IL-6, IL-10, IL-13, and lipid-derived factors [184]. Consistent with the broader concept of macrophage plasticity in liver pathology [185], these signals skew infiltrating monocytes toward an M2-like phenotype. In HCC, M2 TAMs display immunosuppressive and pro-angiogenic properties and are associated with poor clinical outcomes [186]. Once polarized, they secrete protumoral mediators including IL-6, TNF-α, CXCL8, VEGF, IL-23, PDGF, MMP2, and PAI-1, thereby activating STAT3- and NF-κB-dependent programs, promoting EMT, enhancing stemness, and facilitating metastasis [184]. Moreover, TAMs recruit regulatory T cells and suppress cytotoxic CD8^+^ T-cell responses, reinforcing immune exclusion.

The interaction between CAFs and TAMs is not merely parallel but synergistic. CAF-induced M2 macrophages further amplify fibroblast activation through TGF-β, PDGF, and related growth factors, establishing a feed-forward loop that sustains fibrosis, angiogenesis, and immune suppression [187]. A key mediator of this reciprocal crosstalk is osteopontin (OPN), secreted by both TAMs and TAM-educated CAFs; OPN enhances cell–matrix interactions, cytokine signaling, and tumor cell migration, while its inhibition significantly restrains HCC proliferation and invasiveness [187,188]. Importantly, macrophage states in HCC do not strictly conform to a binary M1/M2 model but rather exhibit hybrid transcriptional profiles shaped by fibrotic and hypoxic cues, further complicating therapeutic targeting [189].

Translational evidence supports the clinical relevance of this stromal–myeloid axis. In a cohort of 109 patients undergoing curative resection, intratumoral co-enrichment of αSMA^+^ CAFs and CD204^+^ TAMs defined an aggressive TME and independently predicted worse overall survival [190]. The strong correlation between fibroblast and macrophage markers suggests coordinated remodeling of the stromal and myeloid compartments. Notably, tumors with dual enrichment displayed lower diffusion MRI apparent diffusion coefficient (ADC) values, indicating that preoperative ADC measurements may serve as a non-invasive surrogate of a fibrotic, immunosuppressive microenvironment and assist in risk stratification [190].

CD36^+^ CAFs emerge as potent drivers of metabolic–immune crosstalk. Wang and colleagues demonstrated that enhanced uptake of oxidized lipids activates the p38–CEBP pathway, increasing secretion of macrophage MIF. CAF-derived MIF signals through CD74 on myeloid cells, promoting expansion and immunosuppressive polarization of MDSCs and macrophages, elevating iNOS and IL-6 production, activating NF-κB and STAT3 pathways, suppressing cytotoxic CD8^+^ T cells, and reinforcing tumor stemness [40]. This lipid-driven stromal–myeloid circuitry contributes to immune evasion and resistance to immunotherapy. In parallel, FAP^+^ CAFs have been shown to interact with DAB2^+^ macrophages at tumor margins, forming a structural and immunological barrier that restricts CD8^+^ T-cell infiltration and correlates with poor outcomes and reduced response to immunotherapy [191].

Collectively, these findings indicate that the CAF–TAM axis in HCC is a dynamic, self-reinforcing network that integrates matrix remodeling, metabolic reprogramming, and immune suppression. 

### 7.4 CAF–Macrophages Crosstalk in CCA

CCA cells recruit circulating monocytes through MCP-1/CCL2, CSF-1, and VEGF-A, promoting differentiation into M2-skewed TAMs [192]. Nevertheless, although CAFs and TAMs are consistently identified as major and interdependent components of the CCA desmoplastic stroma, a direct and mechanistically defined interaction between these two populations has not been clearly demonstrated to date. The available studies show that CAFs produce chemokines such as CCL2 and other soluble mediators capable of recruiting monocytes into the TME as shown in other cancer types [193] and TAM infiltration correlates with angiogenesis, Treg accumulation, and poor prognosis in CCA [194].

Moreover, signaling axes such as TWEAK/Fn14 appear to coordinate macrophage recruitment and CAF proliferation within the tumor niche [195].

However, these observations primarily support parallel activation or coordinated remodeling of the stromal–myeloid compartment rather than a clearly delineated, causal pathway whereby CAFs directly reprogram TAM phenotype or function in CCA. Thus, while CAF–TAM crosstalk is biologically plausible and strongly suggested, its precise molecular mechanisms and hierarchical organization remain, at present, insufficiently characterized and warrant further dedicated investigation.

### 7.5 CAF–Immune Cell Crosstalk in HCC and iCCA: Established Mechanisms and Open Questions

Taken together these findings show that evidence for CAF–immune-cell crosstalk is more advanced in HCC than in iCCA. In HCC, distinct CAF subsets have been linked to specific immune effects, including myeloid recruitment, T-cell modulation, and response to ICB. For example, CD36^+^ CAFs promote an immunosuppressive microenvironment through MIF-dependent recruitment of CD33^+^ myeloid cells [40]. In contrast FMO2+ CAFs appear to enhance anti-PD-1 responsiveness by promoting CCL19-dependent tertiary lymphoid structure formation and increased infiltration of CD8^+^ T cells and M1-like macrophages [40,140]. These findings indicate that CAFs are functionally heterogeneous and should not be considered uniformly tumor-promoting.

In iCCA, CAF–myeloid crosstalk is biologically relevant but less mechanistically defined. iCCA is characterized by a dense desmoplastic stroma containing diverse CAF subpopulations and abundant immune–stromal interactions [81,144]. Fibroblastic FAP has been shown to activate STAT3–CCL2 signaling and promote recruitment of immunosuppressive myeloid cells, supporting tumor growth and angiogenesis [196]. However, direct evidence that specific iCCA CAF subsets drive TAM polarization remains limited. Thus, whether CAF-derived signals preferentially recruit TAMs or MDSCs, and whether spatial CAF–macrophage proximity reflects functional crosstalk, should be considered unresolved questions.

### 7.6 CAF-Related Liquid Biopsy and Extracellular Vesicle-Based Biomarkers

Beyond tissue-based characterization, CAF-related biomarkers are emerging as promising tools for liquid biopsy and longitudinal disease monitoring [197,198]. While CAF biology has traditionally been investigated in tumor specimens, accumulating evidence indicates that circulating CAFs (cCAFs) and CAF-derived extracellular vesicles (EVs) can provide minimally invasive information on stromal activation, tumor progression, metastatic dissemination, therapeutic resistance, and treatment response [199,200]. 

In liver cancer, emerging evidence highlights the biological and translational relevance of CAF-derived vesicular communication. In HCC, Zhu et al. identified POSTN^+^ CAFs as a major source of migrasomes at the tumor–stroma interface. These migrasomes promote angiogenesis, metabolic reprogramming, DNA damage, immune exclusion, and resistance to immune checkpoint blockade. A high migrasome-related score was also associated with poor prognosis, supporting their potential role as biomarkers and therapeutic targets [201]. These findings support the concept that CAF-derived vesicular signals may function both as mediators of immune escape and as candidate biomarkers of stromal activity and therapeutic resistance.

Beyond their potential role as biomarkers, CAF-derived vesicles are also being investigated as therapeutic tools. Ding et al. developed NGR-modified CAF-derived exosomes loaded with resveratrol to target MDSCs, inhibit CXCR2/NF-κB signaling, alleviate immune suppression, restore CD8^+^ T-cell antitumor activity, and suppress liver cancer progression [202]. Although still at the preclinical stage, these findings illustrate the potential of engineered CAF-derived vesicles as novel platforms for stromal and immune modulation.

Although direct evidence in CCA remains limited, EVs are increasingly recognized as key mediators of intercellular communication within the TME, regulating interactions among cancer cells, stromal cells, and immune populations [203,204]. Through the transfer of proteins, lipids, cytokines, and regulatory RNAs, EVs can influence stromal remodeling, immune evasion, and tumor progression. In several solid malignancies, CAF-derived EVs have been implicated in the establishment of immunosuppressive microenvironments, metastatic dissemination, and therapy resistance, highlighting their potential role in shaping tumor evolution [199,200].

Given the highly desmoplastic and immune-excluded phenotype characteristic of iCCA, circulating CAF-derived vesicles and other stromal biomarkers represent attractive candidates for liquid biopsy-based monitoring of disease progression, treatment response, and therapeutic resistance, although their clinical utility in iCCA remains to be established [197,198,204]. Consistent with this concept, recent evidence from lung cancer has further highlighted the immunoregulatory role of CAF-derived small EVs (sEVs) in resistance to ICB, supporting the broader translational relevance of EV-mediated CAF–immune crosstalk across solid tumors [205].

Overall, liquid biopsy approaches based on cCAFs, CAF-derived exosomes, migrasomes, and other CAF-associated stromal signatures may complement tissue-based spatial and single-cell analyses. However, their clinical implementation in HCC and iCCA will require prospective validation, standardized detection platforms, and integration with emerging stromal biomarkers, including CAF-rich phenotypes, FAPI-PET imaging, immune exclusion signatures, as well as assessment of their association with response to immunotherapy.

The role of CAF-derived EVs in immune remodeling and their emerging potential as liquid biopsy biomarkers in liver cancer are summarized in Fig. 2. 

**Figure 2:**
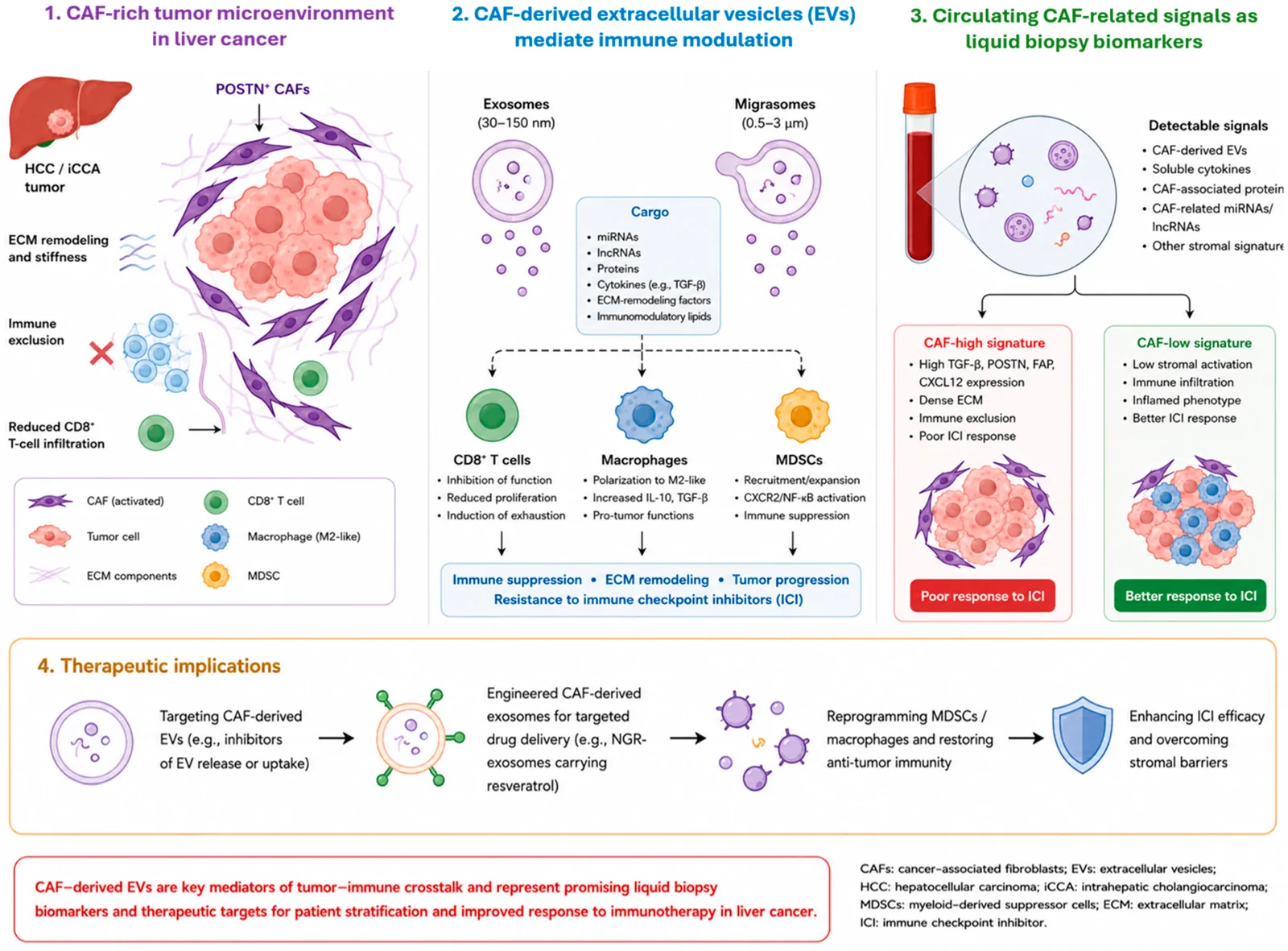
Cancer-associated fibroblast-derived extracellular vesicles and liquid biopsy biomarkers in liver cancer. Cancer-associated fibroblasts (CAFs) contribute to immune remodeling in hepatocellular carcinoma (HCC) and intrahepatic cholangiocarcinoma (iCCA) through extracellular matrix (ECM) remodeling and the release of extracellular vesicles (EVs), including exosomes and migrasomes. CAF-derived EVs modulate immune-cell function and promote resistance to immune checkpoint inhibitors (ICIs). Emerging circulating CAF-associated signals and EV-derived stromal signatures may represent promising liquid biopsy biomarkers for patient stratification and monitoring of CAF-driven immune suppression. Created with AI-assisted illustration tools.

### 7.7 CAF Functional Duality and Stromal Normalization Strategies

Despite the growing interest in CAF-targeted therapies, accumulating evidence indicates that indiscriminate stromal depletion should be approached with caution because of the marked functional heterogeneity and plasticity of CAF populations [15,202,203,204]. 

The cautionary experience in pancreatic ductal adenocarcinoma is particularly instructive. Genetic depletion of α-SMA-positive myofibroblasts resulted in more invasive, poorly differentiated, hypoxic, and immunosuppressed tumors, accompanied by increased regulatory T-cell infiltration and reduced survival [203]. Consistently, genetic deletion of Sonic Hedgehog in pancreatic tumor cells reduced stromal content but generated more aggressive and highly vascularized tumors, demonstrating that selected stromal components may restrain rather than support tumor progression [205]. Independent experiments further demonstrated that both genetic and pharmacological inhibition of Hedgehog signaling suppressed desmoplasia but accelerated pancreatic cancer progression [204].

A comparable functional duality is emerging in liver cancer as well. In HCC, FMO2-positive CAFs have been associated with a favorable clinical outcome and improved response to anti-PD-1 therapy [140]. Mechanistically, this subset promotes CCL19–CCR7-dependent tertiary lymphoid structure formation and enhances the infiltration of CD8-positive T cells and M1-like macrophages [140]. Although these findings require validation in larger independent cohorts, they provide liver-specific evidence that selected CAF populations may support antitumor immunity. Accordingly, CAF-directed treatment should not aim at indiscriminate fibroblast depletion. More appropriate strategies may include selective targeting of tumor-promoting CAF states, inhibition of defined immunosuppressive mediators, normalization of ECM architecture, or reprogramming of CAF plasticity. Several approaches targeting TGF-β, IL-6, CXCL12/CXCR4 signaling, and FAP-associated pathways have demonstrated the potential to enhance antitumor immunity while preserving stromal functions that may restrain tumor progression [15,206,207,208,209,210].

However, the identification of robust biomarkers capable of distinguishing tumor-promoting from tumor-restraining CAF populations remains a major unresolved challenge. Addressing this issue will be critical for the safe clinical implementation of CAF-targeted therapies and for the development of precision stromal interventions in liver cancer.

## 8 Therapeutic Implications and Current Clinical Limitations of CAF Targeting in Primary Liver Cancers

Despite the strong biological rationale for targeting CAF-driven stromal–immune interactions, the clinical translation of these approaches in primary liver cancer remains preliminary. No CAF-selective intervention has yet demonstrated clinical efficacy in late-stage prospective trials specifically conducted in HCC or iCCA. The available human evidence derives mainly from retrospective tissue analyses, CAF-related transcriptional signatures, spatial profiling studies, and early clinical evaluation of pathways involving CAFs together with multiple other cellular compartments of the TME. In iCCA, the evidence supporting direct CAF targeting remains predominantly preclinical or translational, and no CAF-specific therapeutic strategy has yet entered routine clinical practice [211]. Accordingly, the therapeutic implications discussed in this section should be interpreted as hypothesis-generating and as a framework for developing biomarker-driven combination strategies rather than as evidence supporting the clinical use of CAF-directed therapies.

It is additionally important to distinguish direct CAF targeting from the inhibition of pathways in which CAFs participate. Strategies directed against TGF-β, CXCL12–CXCR4, FAP-associated signaling, angiogenesis, or ECM remodeling may indirectly modify CAF functions, but these pathways are not fibroblast-specific and also regulate tumor, endothelial, and immune cells. For example, the TGF-β receptor I inhibitor galunisertib was evaluated in combination with sorafenib in a phase II study of advanced HCC [212]. However, because TGF-β signaling operates across several cellular compartments, this study cannot be considered evidence of CAF-specific clinical efficacy. Thus, the principal current value of CAF research lies in identifying stromal biomarkers of prognosis and therapeutic resistance and in developing rational combinations that selectively disrupt tumor-promoting CAF programs while preserving potentially protective stromal functions.

Therefore, although the potential therapeutic application of CAFs to primary liver cancer lies in a robust rationale based on the role they play as part of TIME, evidence on how to target them, either directly or indirectly, is sparse. Immune checkpoint inhibition in liver cancers involves a broad and evolving therapeutic landscape targeting PD-1/PD-L1 and CTLA-4 axes, with combination regimens (including dual checkpoint blockade and checkpoint inhibition plus anti-angiogenic therapy) [213] emerging as key strategies to counteract the CAF-driven immunosuppressive TME and to overcome both primary and acquired resistance. Importantly, the direct therapeutic targeting of CAFs is complicated by their functional heterogeneity and context-dependent roles. Evidence from experimental models, particularly in pancreatic cancer, has shown that global stromal depletion can paradoxically accelerate tumor progression, revealing that specific CAF subsets may exert tumor-restraining functions [214]. These observations highlight the limitations of non-selective stromal targeting and support a shift toward CAF reprogramming or subset-specific therapeutic strategies in primary liver cancers. In this review, we will cover only the therapeutic strategies that have been attempted in the clinical stage or that are being tested in ongoing clinical trials, since the discussion of pre-clinical evidence taken from *in-vitro* and *in-vivo* settings goes beyond the aim of this paper and has been extensively reviewed elsewhere [29].

Moreover, we will focus on the predictive/prognostic significance of CAFs for the approved therapeutic strategies for BTC, with particular emphasis on iCCA, and HCC: this knowledge may inform a more tailored allocation of treatment following the paradigm of precision medicine.

### 8.1 Predictive Significance of CAFs for Available Treatments: HCC

The relevance of CAFs to the efficacy of different therapeutic strategies in HCC was initially investigated through bioinformatic analyses of publicly available transcriptomic datasets from patient cohorts. Recent studies developed CAF-associated gene signatures to identify related molecular pathways, stratify patients, and generate hypotheses regarding treatment sensitivity. In detail, HCC samples from TCGA and 115 from GEO database were accessed. A high CAFs-scoring caused a trend toward worse survival in the analysed HCC records, despite not statistically significant, suggesting an indirect negative prognostic role of CAFs in the natural history of this disease [215]. Among the genes comprised in the CAFs-signature, those bearing a significant prognostic role for HCC in the above-mentioned dataset functionally belonged to “cell cycle” and “DNA replication” pathways, suggesting these pathways as the potential mechanisms via which CAFs mediate HCC progression. Of note, high CAFs-scoring samples showed a higher potential for immune escape according to tumor immune dysfunction and exclusiontumor (TIDE) analysis and a significantly different drug-sensitivity compared to low CAFs-scoring samples: of these, sorafenib was among the drugs showing a reduced sensitivity, while 4 drugs (out of 193) were identified as potentially more effective in high CAFs-scoring HCCs. These results are in line with 2023 data [216] that described increased resistance to sorafenib when HCC and normal fibroblasts co-cultured occurred in presence of HCC-sorted CAFs, via CXCL12 secretion.

Moving from these preliminary results, the spatial transcriptomic analyses of the neoadjuvant prospective CaboNivo trial (NCT03299946) [217] have recently given the most translationally sound results. Although cabozantinib plus nivolumab is not currently established as a standard therapeutic strategy for HCC, these analyses inform on the putative role of CAFs on immunotherapy and anti-angiogenics sensitivity and resistance. In detail, the authors [217] compared the ST profiles of 4 responders (i.e., achieving major or complete pathological response at surgery) vs. 3 non-responder patients. Responders’ tissues were characterized by an increased presence of immune cells and by an active immune or inflammatory response at genomic-signature level. Specifically to CAFs clusters, they were identified in all 4 responders, whilst only in 1 non-responder, with a specific spatial localization and gene expression: CAFs surrounding the FAP inhibitor positron emission tomography (FAPI-PET) tumor spots were enriched in FOS and JUN expressions that are markers of ECM remodelling. This is suggested by the authors to be “*the result of fibrosis as a response to cell death that recruits CAFs and initiates immune exclusion in collagen/CAF-rich regions and so creates a niche characterized by a lack of immune cells*”, thus opening up to the rationale to test CAFs inhibiting drugs in combination with immunotherapy. In addition, the presence of CAFs might help explain the heterogeneous behavior of responders after surgery: the tumour of the patient who relapsed earlier than the others was more enriched in immune-poor regions characterized by FOS/JUN/CAFs activation and stemness-like genomic signature. These findings should be interpreted as exploratory, given the limited sample size and hypothesis-generating nature of the study.

The potential influence of CAFs on immunotherapy should be considered within the broader and rapidly evolving ICI landscape. In advanced HCC, randomized phase III trials support biologically distinct ICI-based strategies, including combined PD-L1 and VEGF inhibition with atezolizumab plus bevacizumab [218], dual CTLA-4 and PD-L1 blockade with the STRIDE regimen of tremelimumab plus durvalumab [219], and combined PD-1 and CTLA-4 blockade with nivolumab plus ipilimumab [220].

If selective CAF-targeted strategies are not ready for primetime as previously discussed, they should be considered in a biomarker-driven framework, in the context of already available therapeutic classes for primary liver cancers.

Inhibition of CAF-related pathways provides a strong rationale for combination with anti-angiogenic and immune checkpoint therapies, as these strategies may synergistically facilitate lymphocyte infiltration and activation while dismantling extracellular-matrix barriers [134].

The most recent rationale for a combination strategy is given by the epigenetic metabolic reprogramming exerted by CAFs in HCC, leading to the increase in PD-L1-mediate immune evasion [216]. This evidence is built from a translational approach moving from patient derived CAFs, organoids and RNA sequencing data which identifies high nicotinamide N-methyltransferase (NNMT) in CAFs as the key mechanistic determinant of immunotherapy resistance tested *in vivo*, and NNMT-ANGPTL4 the axis to be targeted in order to restore CD8^+^ T-cell activity, thus immunotherapy sensitivity. In addition to this mechanistic evidence, in this setting, anti-angiogenic agents or multi-kinase inhibitors may contribute to stromal and vascular normalization, potentially further improving T-cell infiltration and ICI sensitivity, as also suggested by preclinical evidence showing that lenvatinib combined with anti-PD-1 therapy can activate CD8^+^ T cells through reduction of tumor-associated macrophages and activation of interferon-related pathways [221].

Secondly, tumors characterized by TGF-β-activated myofibroblastic CAFs, collagen-rich immune-excluded stroma, or POSTN/FAP-high programs may be candidates for combined TGF-β/CAF pathway inhibition and PD-1/PD-L1 blockade, based on evidence that TGF-β signaling in stromal fibroblasts promotes T-cell exclusion and resistance to checkpoint inhibition [218].

Thirdly, CAF-subset-directed approaches, such as targeting FAP+ stromal programs or CD36^+^ immunosuppressive CAFs, may be integrated with ICIs or TKIs to reduce myeloid-mediated immunosuppression and improve antitumor immunity; in particular, CD36^+^ CAFs have been shown to promote an immunosuppressive HCC microenvironment through secretion of macrophage MIF [219].

Although these regimens differ in their immunological, vascular, and cytotoxic components, CAF-associated ECM barriers, TGF-β and CXCL12 signaling, recruitment of immunosuppressive myeloid populations, and T-cell exclusion may represent shared mechanisms of primary or acquired resistance. Nevertheless, direct clinical evidence linking a defined CAF subset or stromal signature to differential benefit from a specific checkpoint-inhibitor regimen remains limited. CAF-related signatures should therefore currently be regarded as candidate biomarkers for prospective patient stratification rather than as validated tools for selecting a particular immunotherapy.

### 8.2 Predictive Significance of CAFs for Available Treatments: BTC

At present, no published papers have suggested a role for infiltrated CAFs in first line chemo-immunotherapy treatment sensitivity or resistance in this disease. Most of the mechanistic evidence of the negative prognostic role of CAFs is derived from pre-clinical studies where the pathogenic role of this cellular subtype is explored *in vitro* [222,223]. Its closest translation to the clinical setting derives from the *ex-vivo* studies of CAFs on clinical samples, conducted since 2022, which will be reviewed hereafter.

Qiqi Cao et al. published a transcriptomic dissection of 14 iCCA samples and their adjacent non-tumoral tissues of treatment-naive patients, performed with a scRNA-seq approach [224]. The authors described the high intrinsic heterogeneity of different cell clusters, comprising those belonging to TME. However, LGALS1^+^ fibroblastsrepresented the preferably enriched population in tumor tissue and actively interacted with cholangiocytes to drive a malignant phenotype.

Bao-Ye Sun et al. described the peculiar scRNA-seq differences between ICI-treated iCCA patient samples and non-ICI treated ones [225]. They showed that ICI exposure caused an increase in the number and strength of the interactions between malignant cholangiocytes and CAFs, and that CAFs seem to mediate the promotion of angiogenesis after ICI exposure. Despite not being directly translatable as a clinical application, this observation suggests the rationale to target CAFs and angiogenesis as a rescue strategy to overcome secondary resistance to ICI in iCCA.

Furthermore, in iCCA, where FAP expression is particularly enriched in the desmoplastic stroma, FAPI-PET may help identify FAP-high tumors and guide future theranostic or combination strategies, as supported by the superior tumor detection of 68Ga-FAPI-46 compared with 18F-FDG PET/CT and conventional CT in CCA [226]. Overall, CAF-high/FAPI-positive, immune-excluded, angiogenic, or myeloid-enriched tumors may be prioritized for CAF-targeted combinations, whereas CAF-low tumors or tumors enriched in immune-supportive CAF subsets may require different strategies to avoid unnecessary depletion of potentially protective stromal components.

### 8.3 Diagnostic and Theranostic Applications of FAP Targeting

The diagnostic, and possibly theranostic, application of CAFs seems closer to its clinical application. In particular, FAP inhibitor positron emission tomography (FAPI-PET) has been developed from the discovery that FAP is overexpressed by CAFs, proving a higher sensitivity and specificity in gastro-intestinal cancers detection and treatment monitoring [227]. Moving from these considerations, prospective clinical trials designed to tailor FAPI-PET application to gastrointestinal cancers (comprising HCC) are ongoing (NCT05641896, NCT05687747) and early positive reports have been recently presented showing a high positive predictive value (PPV) for identifying FAP expression [228].

However, increased FAPI uptake does not establish that elimination of FAP-positive CAFs will improve clinical outcomes. FAP can be expressed by functionally heterogeneous stromal populations, and imaging does not currently distinguish tumor-promoting from potentially tumor-restraining CAF states.

FAPI-PET may be particularly valuable for the evaluation of primary liver tumors because of the low physiological uptake of several FAPI tracers in normal liver tissue and the variable sensitivity of ^18^F-fluorodeoxyglucose positron emission tomography in HCC and [229]. In a comparative study of primary liver malignancies, FAPI-PET/CT demonstrated improved lesion detection and tumor-to-background contrast compared with FDG-PET/CT, although the available cohorts were limited and heterogeneous [230]. These findings support further prospective evaluation of FAPI-PET for HCC detection, staging, and treatment monitoring, but they do not yet justify its routine use as a replacement for established contrast-enhanced imaging modalities.

FAPI-based imaging is also of interest in BTC, which is frequently characterized by abundant FAP-expressing desmoplastic stroma. In an early pan-cancer analysis, biliary tract tumors showed high FAPI tracer uptake, although the number of cases was small [231]. More recently, a systematic review and meta-analysis reported superior diagnostic performance and greater tumor uptake for ^68^Ga-FAPI PET/CT compared with ^18^F-FDG PET/CT in BTC, particularly for lymph-node and distant-metastasis detection [232]. However, methodological heterogeneity, limited cohort sizes, and the absence of standardized acquisition and interpretation criteria remain important limitations.

FAP also represents a potential theranostic target because the same molecular scaffold can be labeled with diagnostic or therapeutic radionuclides. Nevertheless, FAP-directed radioligand therapy remains investigational in primary liver cancer. Its development will require prospective assessment of dosimetry, toxicity, treatment efficacy, and the potential effects of targeting FAP-positive stromal populations in non-tumoral tissues. Future studies should correlate FAPI uptake with histological FAP expression, spatial CAF phenotypes, immune-cell distribution, treatment response, and survival. This integration will be essential to establish whether FAPI-PET functions only as a stromal imaging biomarker or can also identify patients with a therapeutically actionable FAP-rich TME.

## 9 Conclusions

CAFs are central regulators of the TIME in HCC and CCA, displaying marked transcriptional, spatial, and functional heterogeneity [29,75,132]. The current body of evidence is more extensive for HCC than for CCA/iCCA, reflecting differences in research maturity rather than biological importance. Importantly, the emerging data in CCA are consistent with the core paradigms established in HCC, supporting the concept of conserved CAF-driven immune remodeling across primary liver cancers, albeit with distinct levels of mechanistic resolution.

CAFs actively promote immune evasion through multiple complementary mechanisms, including physical exclusion of T cells, modulation of chemokine networks that impair lymphocyte recruitment, and direct induction of T-cell dysfunction. In addition, their crosstalk with tumor-associated macrophages reinforces immunosuppressive circuits within the TIME [40,148,174].

Although these functions are well characterized in HCC, emerging evidence indicates that CAFs also play a relevant, though less defined, immunomodulatory role in CCA [180,182]. Importantly, the coexistence of tumor-promoting and potentially tumor-restraining CAF subsets highlights the need for selective targeting or reprogramming strategies rather than indiscriminate stromal depletion.

Overall, a deeper understanding of CAF heterogeneity supports the development of combinatorial therapeutic approaches integrating stromal targeting with immunotherapy to overcome resistance and improve clinical outcomes in primary liver cancers.

Future studies should aim to elucidate the dynamic interactions between CAF subsets and other immune or stromal components over time, enabling the design of precision interventions that selectively enhance anti-tumor immunity while minimizing adverse effects on normal tissue homeostasis. Such insights could pave the way for personalized, stroma-informed therapies in both HCC and CCA.

## Data Availability

Not applicable.

## Abbreviations

The following abbreviations are used in this manuscript:

ADC	apparent diffusion coefficient
AFP	alpha-fetoprotein
apCAFs	antigen-presenting cancer-associated fibroblasts
BTC	biliary tract cancers
CAFs	cancer-associated fibroblasts
CCA	cholangiocarcinoma
CD	cluster of differentiation
circRNAs	circular RNAs
CTGF	connective tissue growth factor
CTLA-4	cytotoxic T-lymphocyte-associated protein 4
CXCL	C-X-C motif chemokine ligand
ECM	extracellular matrix
eCAFs	epithelial–mesenchymal transition-like cancer-associated fibroblasts
EGFR	epidermal growth factor receptor
EMT	epithelial–mesenchymal transition
FAP	fibroblast activation protein
FAPI-PET	fibroblast activation protein inhibitor positron emission tomography
FDG-PET	fluorodeoxyglucose positron emission tomography
FGFR2	fibroblast growth factor receptor 2
FSP-1	fibroblast-specific protein 1
GEO	Gene Expression Omnibus
HBV	hepatitis B virus
HCC	hepatocellular carcinoma
HCV	hepatitis C virus
HPCs	hepatic progenitor cells
HSCs	hepatic stellate cells
IBD	inflammatory bowel disease
ICB	immune checkpoint blockade
ICIs	immune checkpoint inhibitors
IDH1	isocitrate dehydrogenase 1
iCAFs	inflammatory cancer-associated fibroblasts
iCCA	intrahepatic cholangiocarcinoma
IL	interleukin
ITGA6	integrin alpha 6
LAMA4	laminin subunit alpha 4
LSEC	liver sinusoidal endothelial cells
MAFLD	metabolic dysfunction-associated fatty liver disease
mCAFs	matrix cancer-associated fibroblasts
MCP-1	monocyte chemoattractant protein-1
MDSCs	myeloid-derived suppressor cells
MHC-II	major histocompatibility complex class II
miRNA/miR	microRNA
MMP	matrix metalloproteinase
MRI	magnetic resonance imaging
MSCs	mesenchymal stromal cells
MSI	microsatellite instability
MASH	metabolic dysfunction-associated steatohepatitis
myCAFs	myofibroblastic cancer-associated fibroblasts
NF-κB	nuclear factor kappa B
NK	natural killer
NKT	natural killer T
OS	overall survival
PD-1	programmed cell death protein 1
PD-L1/PD-L2	programmed death-ligand 1/2
PDGF	platelet-derived growth factor
PDGFR	platelet-derived growth factor receptor
PFs	portal fibroblasts
PlGF	placental growth factor
POSTN	periostin
PSC	primary sclerosing cholangitis
RGS5	regulator of G protein signaling 5
scRNA-seq	single-cell RNA sequencing
STAT	signal transducer and activator of transcription
ST	spatial transcriptomics;
SUV	standardized uptake value
TAMs	tumor-associated macrophages
TCGA	The Cancer Genome Atlas
TGF-β	transforming growth factor beta
TIDE	tumor immune dysfunction and exclusion
TIME	tumor immune microenvironment
TME	tumor microenvironment
TKIs	tyrosine kinase inhibitors
TLS	tertiary lymphoid structures
TNF-α	tumor necrosis factor alpha
Tregs	regulatory T cells
VEGF	vascular endothelial growth factor
vCAFs	vascular cancer-associated fibroblasts
